# Supplementary material for: Type I interferon shapes brain distribution and tropism of tick-borne flavivirus
Source: Nat Commun. 2023 Apr 10;14:2007. doi: 10.1038/s41467-023-37698-0 (PMC10086010; doi:10.1038/s41467-023-37698-0)
Supplement: Supplementary file 1 — Supplementary Information [file 41467_2023_37698_MOESM1_ESM.pdf]

Supplementary information

Type I interferon shapes brain distribution and tropism of tick-borne flavivirus

Nunya Chotiwan[1,2,3*,#], Ebba Rosendal[1,2,#], Stefanie M. A. Willekens[1,2,4,#], Erin Schexnaydre[1,2,5,6,7], Emma Nilsson[1,2], Richard Lindqvist[1,2], Max Hahn[4], Ionut Sebastian Mihai[2,8,9], Federico Morini[4], Jianguo Zhang[1,2,5,6,7], Gregory D. Ebel[10], Lars-Anders Carlson[2,5,6,7], Johan Henriksson[2,7,8] , Ulf Ahlgren[4], Daniel Marcellino[11], Anna K. Överby[1,2*]

[1] Department of Clinical Microbiology, Umeå University; 90185 Umeå, Sweden

[2] The Laboratory for Molecular Infection Medicine Sweden (MIMS), Umeå University; 90187 Umeå, Sweden

[3] Chakri Naruebodindra Medical Institute, Faculty of Medicine Ramathibodi Hospital, Mahidol University, Samut Prakan, 10540, Thailand

[4] Umeå Centre for Molecular Medicine (UCMM), Umeå University; 90187 Umeå, Sweden

[5] Wallenberg Centre for Molecular Medicine, Umeå University; 90187 Umeå, Sweden

[6] Department of Medical Biochemistry and Biophysics, Umeå University; 90187 Umeå, Sweden

[7] Umeå Centre for Microbial Research, Umeå University; 90187 Umeå, Sweden

[8] Department of Department of Molecular biology, Umeå University; 90187 Umeå, Sweden

[9] Företagsforskarskolan, Umeå University, 90187 Umeå, Sweden

[10] Department of Microbiology, Immunology and Pathology, Colorado State University, USA

[11] Department of Integrative Medical Biology, Umeå University; 90187 Umeå, Sweden

*Corresponding authors: Anna K Överby and Nunya Chotiwan
[#] Contributed equally

Email: anna.overby@umu.se and nunya.chotiwan@umu.se

This PDF file includes:

Supplementary figures 1-5

Supplementary Table 1

Other supplementary materials for this manuscript include the following:

Supplementary Movie 1-3

Supplementary Data 1-2

Source Data file

Supplementary figures

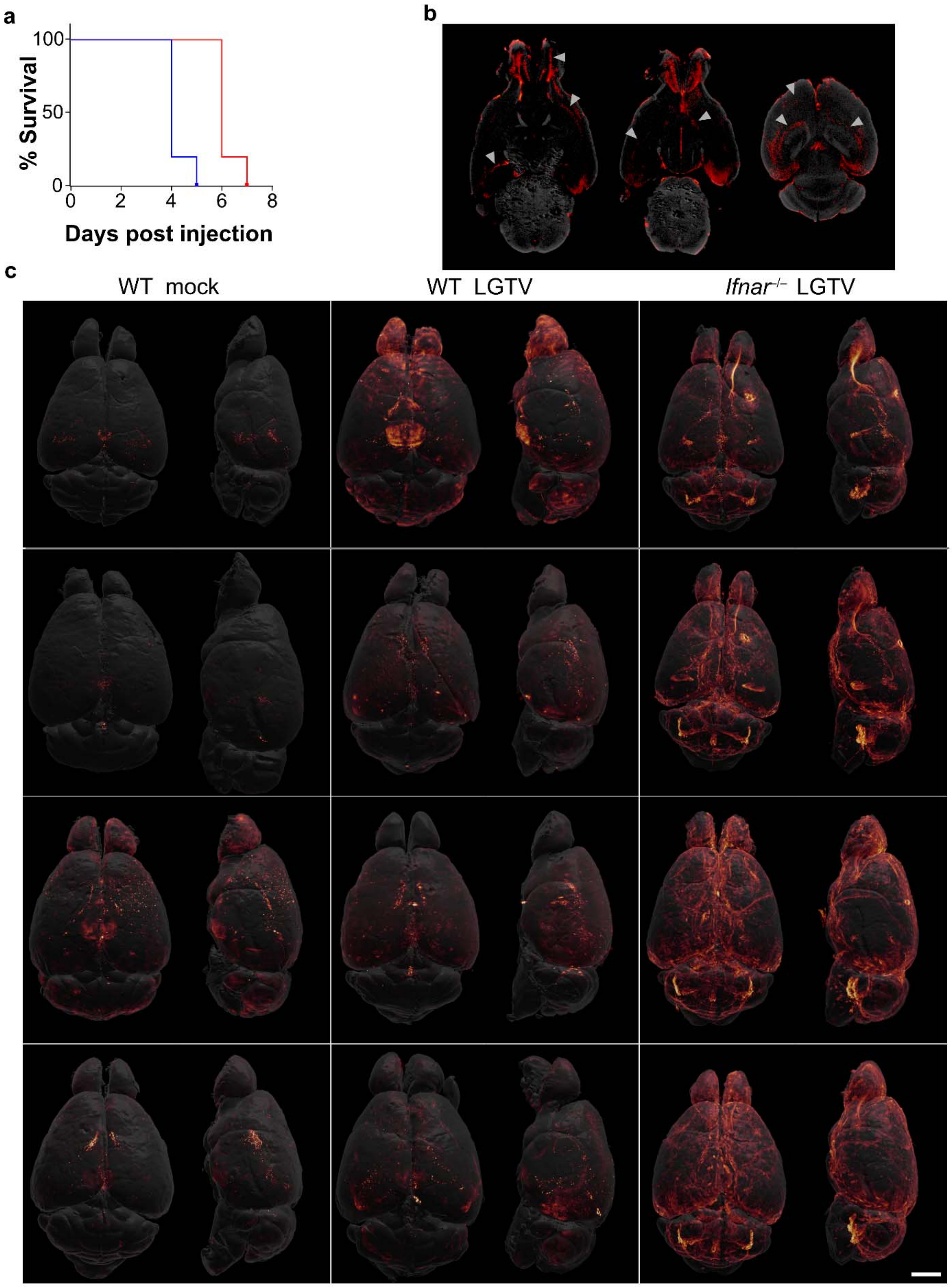

Supplementary Fig. 1. IFN-I response influences host survival and distribution of LGTV infection. a Survival analysis of WT and *Ifnar*$^{-/-}$ mice, intracranially injected with 1000 pfu of LGTV (n = 5). Survival differences between groups were significant (log-rank test, p = 0.0031). **b** OPT cross sections of a representative *Ifnar*$^{-/-}$ brain showing antibody penetration in the deeper areas of the brain (arrows). **c** OPT-scanned immunolabeled brain reveals the distribution of LGTV infection in the adult mouse brain. Volumetric 3D render of supplementary OPT scans of the brain from mock and LGTV infected mice immunolabeled with antibodies against viral NS5. The signal intensity was normalized within an individual brain and adjusted to identical minimum and maximum. The viral signal was overlaid onto the anatomical outlines created from iso-surface rendering of the autofluorescence signal of each brain. For each image pair, the top and lateral views of the same specimen are shown. Scale bar = 2000 µm. Source data are provided as a Source Data file.

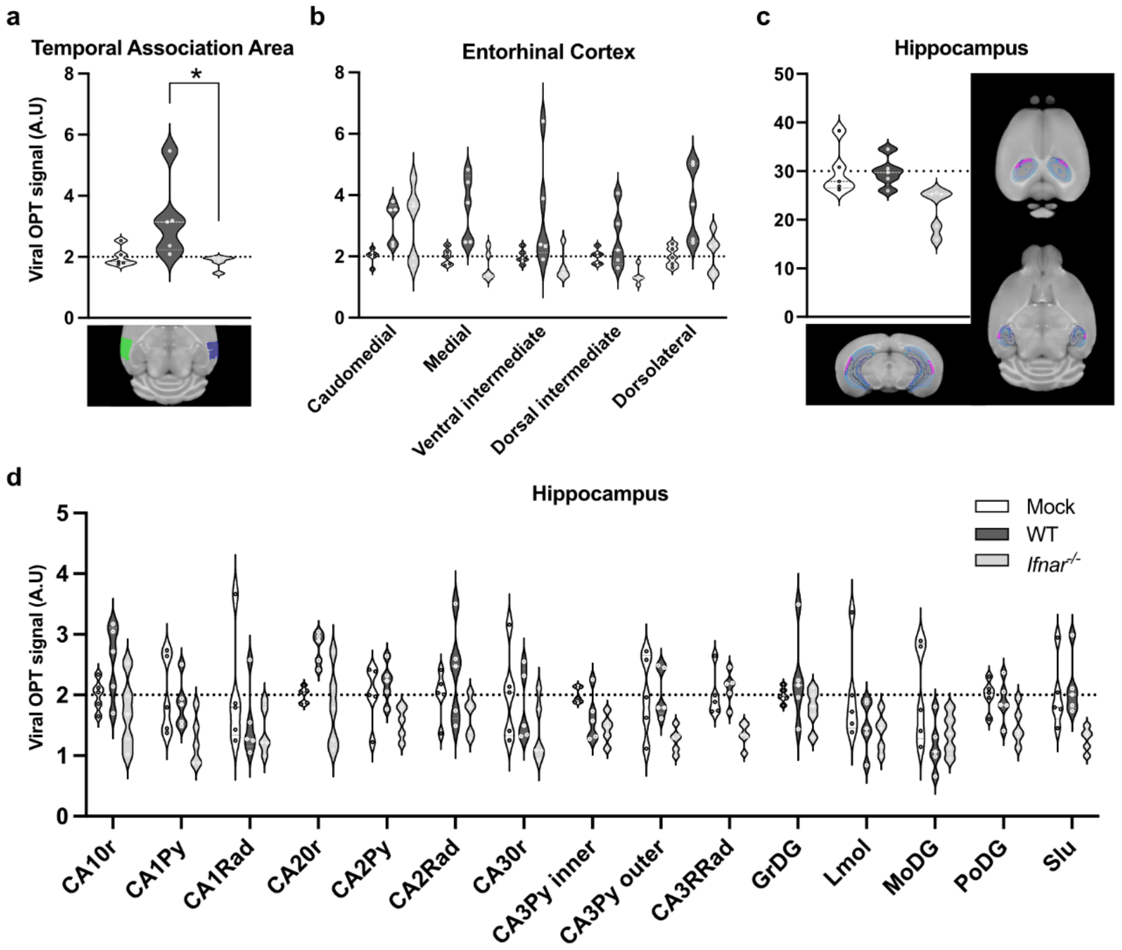

Supplementary Fig. 2. Image and quantification of viral infection in cerebral cortex regions. Quantification and statistical analysis (two-tailed unpaired t-test (a and c) with Holm-Sidak multiple comparison correction (c and d) of viral OPT signal in **a** temporal association area (*p=0.047), **b** individual VOIs in the composite entorhinal cortical region, **c** composite hippocampal region, and **d** individual VOIs in the hippocampal region. Source data are provided as a Source Data file.

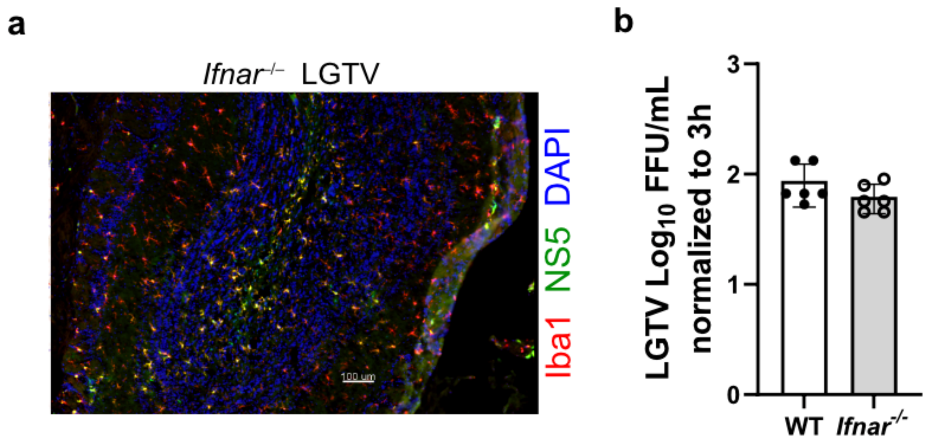

Supplementary fig. 3 Microglia staining of OB and primary microglia infected with LGTV *in vitro*. a) Maximum-intensity projection of confocal z-stack. The images were taken from sagittal brain sections (10 µm) using confocal microscope, representative picture of OB is shown. Scale bars = 100 µm. The sections were immunolabeled using anti-NS5 (green), anti-Iba1 (red) for microglia, and DAPI for nucleus (blue). b) Primary microglia were isolated from WT and *Ifnar*$^{-/-}$ brains. The cells were infected with LGTV (MOI 1) for 72 h and viral growth was measured by focus forming assay, bars show mean ± SD (n = 6). Source data are provided as a Source Data file.

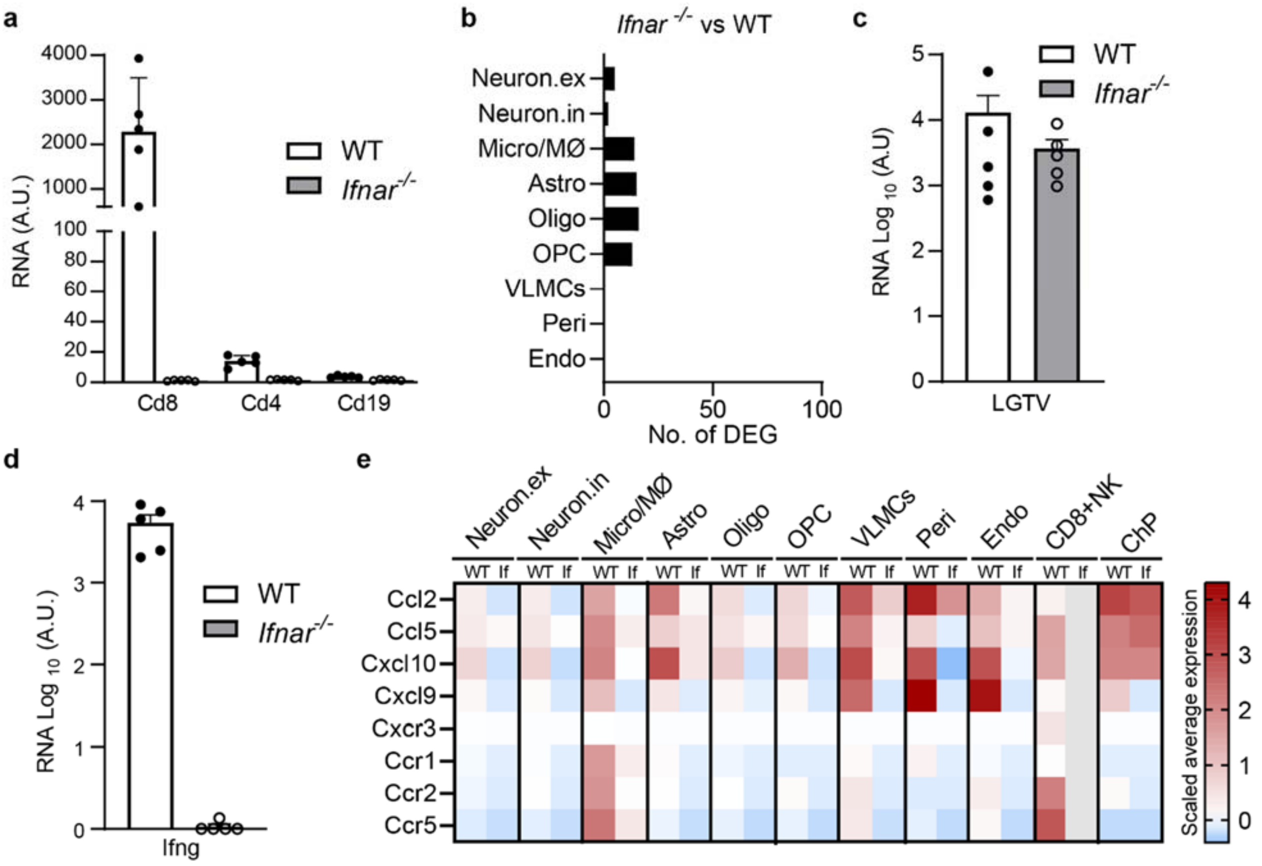

Supplementary Fig. 4. Transcriptional differences and viral load in WT and *Ifnar*$^{-/-}$ mice. a Gene expression of *Cd8*, *Cd4* and *Cd19* in cortex of infected WT (n=5) and *Ifnar*$^{-/-}$ (n=5) mice. Quantified by qPCR and normalized to housekeeping gene *Gapdh,* bars show mean ±SD. **b** Number of DEGs (log2FC>1, padj < 0.05) between *Ifnar*$^{-/-}$ and WT mice in uninfected samples. **c** Viral load in cerebral cortex at the humane endpoint in WT (n=5) and *Ifnar*$^{-/-}$ mice (n=5) infected with 10,000 PFU intracranially. Quantified by qPCR and normalized to housekeeping gene *Gapdh*. Bars represent mean ±SEM. **d** Gene expression of *Cd8*, *Cd4* and *Cd19* in cortex of infected WT (n=5) and *Ifnar*$^{-/-}$ (n=5) mice, bars show mean ±SEM. Quantified by qPCR and normalized to housekeeping gene *Gapdh*. **e** Heatmap showing expression of T cell chemoattractants and corresponding receptors upon infection in WT and *Ifnar*$^{-/-}$ (If) mice. Source data are provided as a Source Data file.

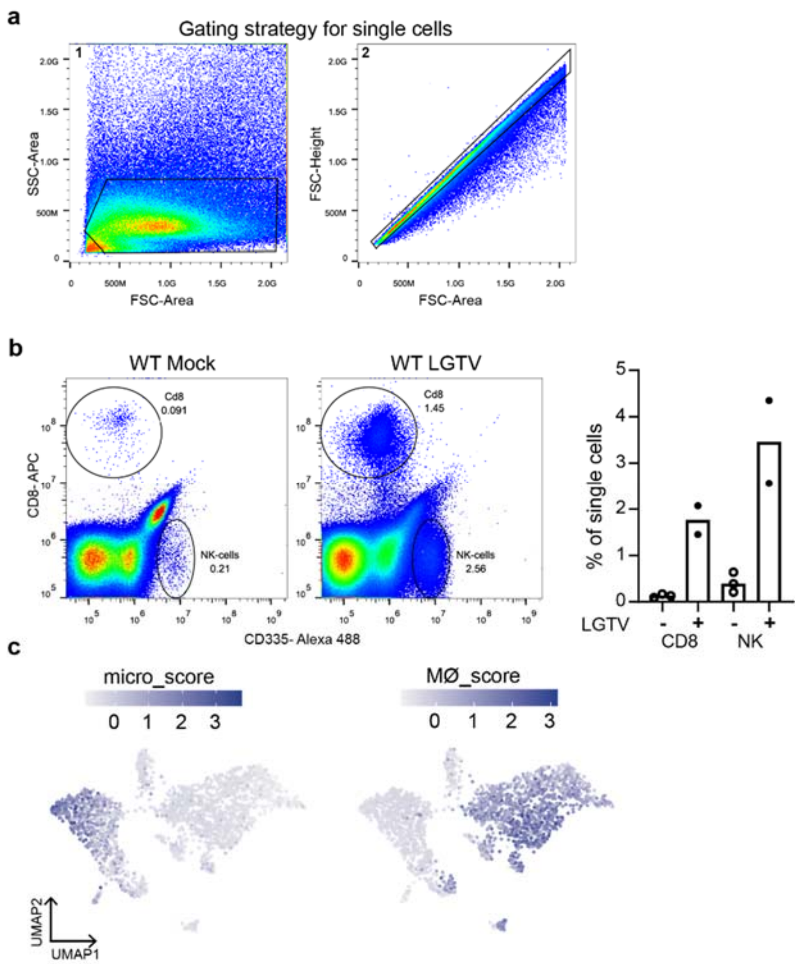

Supplementary Fig. 5. Infiltration of immune cells in WT and *Ifnar*$^{-/-}$ mice. a Representative scatterplots showing gating strategy based on forward scatter (FSC) and side scatter (SSC) for obtaining single cells used in downstream analysis of brain leucocytes. **b** Flow cytometry of brain leukocytes from the cerebral cortex of untreated and infected WT mice. Representative scatterplots and percentage CD8+ T cells and NK cells (CD335^{+}). Boxes represent means of untreated (n=3) and LGTV infected (n=2). **c** Subclustering of 2,363 nuclei of micro/MØ cell subset belonging to all datasets, colored by gene signatures for microglia (*Cx3cr1, P2ry12, Slco2b1, Tmem119*) or MØ (*Slfn4, Ms4a8a, Clec4e, Itga4*) (PMID: 31325960). Source data are provided as a Source Data file.

Supplementary Table 1. Key Antibody Table

Antibodies	Origin	Clonality	Clone name	Working dilution	Use in the current study	Company (Cat#)
**Anti-chicken Alexa Fluor 488**	Goat	Polyclonal	-	1:400	IHC	Thermo Fisher Scientific (A11039)
**Anti-chicken Alexa Fluor 555**	Goat	Polyclonal	-	1:500	IHC	Thermo Fisher Scientific (A21437)
**Anti-chicken Alexa Fluor 680**	Goat	Polyclonal	-	1:500	OPT, IHC	Abcam (ab175779)
**Anti-mouse Alexa Fluor 647**	Goat	Polyclonal	-	1:500	IHC	Abcam (ab150115)
**Anti-rabbit Alexa Fluor 488**	Donkey	Polyclonal	-	1:500	IHC	Thermo Fisher Scientific (A21206)
**Anti-rabbit Alexa Fluor 594 (pre-absorbed)**	Donkey	Polyclonal	-	1:500	OPT	Abcam (ab150064)
**Anti-mouse CD45 Alexa 488**	Rat	Monoclonal	30F-11	1:100	IHC	Thermo Fisher Scientific (53-0451-82)
**AQPI**	Mouse	Monoclonal	sc-32737	1:50	IHC	Santa Cruz Biotechnology (sc-32737)
**CD8**	Rat	Monoclonal	Clone 53-6.7 (RUO)	1:200	IHC	BD Pharmigen (561093)
**CD335**	Rat	Monoclonal	29A1.4	1:500	IHC	Thermo Fisher Scientific (AB_1724164
**Doublecortin**	Rabbit	Polyclonal	-	1:800	IHC	Cell Signaling (4604)
**Iba1**	Rabbit	Polyclonal	-	1:400	IHC	Histolab (CP290)
**NS5***	Chicken	Polyclonal	-	1:1000	OPT, IHC	Agrisera AB
**TMEM119**	Rabbit	Monoclonal	28-3	0.5µg/ml	IHC	Abcam (ab209064)
**TUBB3**	Rabbit	Polyclonal	-	1:3000	IHC	BioLegend (PRB-435P)

*Affinity-purified NS5 antibody was produced in chicken, according to the manufacturer's protocol, using the following peptide sequence of NS5 from tick-borne encephalitis virus strain Torö (GenBank: DQ401140): (carboxylated)-CMDRHDLHWELRLESS-(amidated).
