# Supplementary material for: Type I interferon shapes brain distribution and tropism of tick-borne flavivirus
Source: Nat Commun. 2023 Apr 10;14:2007. doi: 10.1038/s41467-023-37698-0 (PMC10086010; doi:10.1038/s41467-023-37698-0)
Supplement: Supplementary file 3 — Description of Additional Supplementary Files [file 41467_2023_37698_MOESM3_ESM.pdf]

Description of Additional Supplementary Files

File Name: Supplementary Data 1
Description: Quantification of OPT signal

File Name: Supplementary Data 2
Description: Lists of differentially expressed genes, Reactome pathways and cell-cell communication.

File Name: Supplementary Movie 1
Description: **3D renderings of OPT-scanned immunolabeled mouse brain showing the distribution of LGTV infection**. The brains from LGTV infected mice were harvested at endpoint, immunolabeled with anti-NS5 antibody (red glow: normalized to the virus signal intensity within an individual brain) and imaged using OPT.

File Name: Supplementary Movie 2
Description: **LSFM showing high-resolution image of the fourth ventricle ChP infected with LGTV.** (A) Orientation of the brain in the LSFM during image acquisition. The illustration was created with BioRender.com. (B) Volumetric 3D render of LSFM of LGTV infected fourth ventricle ChP, immunolabeled with anti-NS5 antibodies (red glow). ChP was imaged in 3 tiles at 2.5× magnification and stitched together with 20% overlap. Z = 2000 µm; scale bar = 300 µm. Solid-line square indicates the location imaged in (C). Dashedline square shows the orientation of (C). (C) Tomographic section of (B) viewed from the YZ plane at 4× magnification. z = 600 µm.

File Name: Supplementary Movie 3
Description: **FIB-SEM volume image of LGTV infected ChP.** Slice-through view of the FIB-SEM volume image with 3D segmented representation of the volume. Segmentation includes replication complexes (yellow), ER (blue), mitochondria (green), and plasma membranes (pale and dark orange).
