# Supplementary material for: Type I interferon shapes brain distribution and tropism of tick-borne flavivirus
Source: Nat Commun. 2023 Apr 10;14:2007. doi: 10.1038/s41467-023-37698-0 (PMC10086010; doi:10.1038/s41467-023-37698-0)
Supplement: Supplementary file 9 — Reporting Summary [file 41467_2023_37698_MOESM9_ESM.pdf]

nature portfolio

Corresponding author(s): Anna K Överby

Last updated by author(s): Mar 23, 2023

Reporting Summary

Nature Portfolio wishes to improve the reproducibility of the work that we publish. This form provides structure for consistency and transparency in reporting. For further information on Nature Portfolio policies, see our Editorial Policies and the Editorial Policy Checklist.

Statistics

For all statistical analyses, confirm that the following items are present in the figure legend, table legend, main text, or Methods section.

n/a	Confirmed	
☐	☒	The exact sample size (*n*) for each experimental group/condition, given as a discrete number and unit of measurement
☐	☒	A statement on whether measurements were taken from distinct samples or whether the same sample was measured repeatedly
☐	☒	The statistical test(s) used AND whether they are one- or two-sided<br>*Only common tests should be described solely by name; describe more complex techniques in the Methods section.*
☒	☐	A description of all covariates tested
☐	☒	A description of any assumptions or corrections, such as tests of normality and adjustment for multiple comparisons
☐	☒	A full description of the statistical parameters including central tendency (e.g. means) or other basic estimates (e.g. regression coefficient) AND variation (e.g. standard deviation) or associated estimates of uncertainty (e.g. confidence intervals)
☐	☒	For null hypothesis testing, the test statistic (e.g. *F*, *t*, *r*) with confidence intervals, effect sizes, degrees of freedom and *P* value noted<br>*Give P values as exact values whenever suitable.*
☒	☐	For Bayesian analysis, information on the choice of priors and Markov chain Monte Carlo settings
☒	☐	For hierarchical and complex designs, identification of the appropriate level for tests and full reporting of outcomes
☒	☐	Estimates of effect sizes (e.g. Cohen's *d*, Pearson's *r*), indicating how they were calculated

Our web collection on statistics for biologists contains articles on many of the points above.

Software and code

Policy information about availability of computer code

Data collection	Auto Slice and View software (v4.1.1.1582, Thermo Fischer Scientific)<br>Everest v.3.1.18.0<br>Leica Application Suit X software (LAS X, v.3.5.5, Leica)<br>Paravision 7.0 software (Bruker)<br>StepOnePlus real-time PCR system (v2.3, Applied Biosystems)<br>Zeiss Zen interface (v.14.0.19.201, Zeiss)
Data analysis	Amira-Avizo software (v2020.3.1, Thermo Fisher Scientific)<br>Everest v.3.1.18.0<br>CellChat v 1.6.1 (https://github.com/sqjin/CellChat)<br>FlowJo (v10.0.7r2, BD)<br>GraphPad Prism (v9.3.1 495 GraphPad Software Inc.)<br>ImageJ (v. 1.8.0_172, NIH) with linear stack alignment, with SIFT and MultiStackRegistration plugins<br>Imaris (v. 9.7.2, Bitplane)<br>Imaris (v9.5.1, Bitplane)<br>Imaris file converter (v. 9.8.0, Bitplane)<br>Imaris file converter (v9.5.1, Bitplane)<br>Imspector Pro software (v7.0124.0, LaVision Biotec Gmbh)<br>Leica Application Suit X software (LAS X, v.3.5.5, Leica)<br>Matlab (R2014a, The MathWorks 455 Inc., USA).

NRecon software (v.1.7.0.4, Skyscan microCT, Bruker)
PMOD VIEW tool (v.4.2, 460 PMOD Technologies Inc., Switzerland)
PMOD VIEW tool or Amira-Avizo software (v6.3.0, 466 Thermo Fisher Scientific)
TeraStitcher script (v9) implemented in Imspector Pro (LaVision Biotec Gmbh)
The toolbox SPMmouse in SPM8
R software environment (v.4.2.1, The R Foundation) using the Seurat package (v 4.1.0)
RStudio Desktop (v.2022.07.1, RStudio, PBC)
All computer codes used for the snRNAseq analyses are available at https://github.com/ERosendal/LGTV_WT_Ifnar_10x

For manuscripts utilizing custom algorithms or software that are central to the research but not yet described in published literature, software must be made available to editors and reviewers. We strongly encourage code deposition in a community repository (e.g. GitHub). See the Nature Portfolio guidelines for submitting code & software for further information.

Data

Policy information about availability of data

All manuscripts must include a data availability statement. This statement should provide the following information, where applicable:

- Accession codes, unique identifiers, or web links for publicly available datasets
- A description of any restrictions on data availability
- For clinical datasets or third party data, please ensure that the statement adheres to our policy

All data generated or analyzed during this study are provided in the Supplementary Information/Source Data file. Raw single nuclei RNAseq data generated in this study have been deposited in the ArrayExpress database under accession code E-MTAB-12131 . LGTV strain TP21 viral genome sequence is publicly accessible in GenBank (NC_003690). Raw image data can be requested from the corresponding authors with reasonable means to transfer large data files. Source data are provided with this paper.

Human research participants

Policy information about studies involving human research participants and Sex and Gender in Research.

Reporting on sex and gender	n/a
Population characteristics	n/a
Recruitment	n/a
Ethics oversight	n/a

Note that full information on the approval of the study protocol must also be provided in the manuscript.

Field-specific reporting

Please select the one below that is the best fit for your research. If you are not sure, read the appropriate sections before making your selection.

☒ Life sciences ☐ Behavioural & social sciences ☐ Ecological, evolutionary & environmental sciences

For a reference copy of the document with all sections, see nature.com/documents/nr-reporting-summary-flat.pdf

Life sciences study design

All studies must disclose on these points even when the disclosure is negative.

Sample size	The statement indicating the sample size used in each experiment was included in the figure legends. We did not use a statistical method to predetermine the sample size, for the OPT we choose 5 animals per group, this was mainly because we expected to see major differences in distribution patterns between the two genotypes. For the snRNAseq we pooled two animals per condition one male and one female to reduce possible underlining cellular profiling bias due different genders. In accordance with the ethical committee requirements, sample sizes were chosen to limit the use of animals as much as possible while still support meaningful conclusion.
Data exclusions	No data were excluded
Replication	Samples in all experiments were taken from at least 2 individual infection experiments which were performed on different days. In some experiments such as confocal and electron microscope experiments, we included technical replicates for each biological replicate. The statement indicating the replication was included in the figure legends.
Randomization	For virus infection in mice in all experiments, we randomized the infection to the level of the cage but not at the level of individual mice due to the ease of managing. We set the criteria where we used 7-13 weeks-old mice and try to include similar amount of both sexes in each

condition. For brain imaging and analysis, the researcher who was not involved in image analysis gave a code to each brain regardless of different treatments (such as A, B, C,... etc) in random order. The brains were scanned and the images were analyzed by one researcher in random order in a double-blind manner. Lastly, for snRNAseq, we randomly infecting one male and female per condition and pooled the nuclei extracted from both brains.

Blinding

The investigators were not blinded to group allocation during data collection since infected mice showed obvious signs of sickness from virus infection (such as significant weight loss, eye infection, facial edema etc.). However, in the imaging experiments where bias can likely be introduced, we processed the image analysis in a double-blind manner. First, each brain was given an ID that does not indicate the condition of the experiment (such as T1, T2, T3,... etc.). During image analysis, the brain samples were given another code (such as A, B, C,... etc) in random order by the researcher who wasn't involved in the image analysis. Brain images (mock, WT+virus and Ifnar-/- +virus) were analyzed blindly in a random order. The codes associated the samples to the experimental conditions were given after the images were analyzed.

Reporting for specific materials, systems and methods

We require information from authors about some types of materials, experimental systems and methods used in many studies. Here, indicate whether each material, system or method listed is relevant to your study. If you are not sure if a list item applies to your research, read the appropriate section before selecting a response.

Materials & experimental systems

n/a	Involved in the study
☐	☒ Antibodies
☐	☒ Eukaryotic cell lines
☒	☐ Palaeontology and archaeology
☐	☒ Animals and other organisms
☒	☐ Clinical data
☒	☐ Dual use research of concern

Methods

n/a	Involved in the study
☒	☐ ChIP-seq
☐	☒ Flow cytometry
☐	☒ MRI-based neuroimaging

Antibodies

Antibodies used

All antibodies used including species, dilution and cat numbers were provided in the Supplementary table 3 in the Supplementary information file.

Validation

All antibodies used in this paper were extensively validate, in the case of multiple antibody staining the slices were carefully checked for bleed through. For validated of the NS5 antibody we performed immunofluorescence staining of uninfected and infected cells. We also show NS5 staining on mock samples in figure 2 B and D.
For all the other antibodies they are commercially available and several publications are available following these links:
https://www.thermofisher.com/antibody/product/Goat-anti-Chicken-IgY-H-L-Secondary-Antibody-Polyclonal/A-11039
https://www.thermofisher.com/antibody/product/Goat-anti-Chicken-IgY-H-L-Secondary-Antibody-Polyclonal/A-21437
https://www.abcam.com/products/secondary-antibodies/goat-chicken-igy-hl-alexa-fluor-680-ab175779.html
https://www.abcam.com/products/secondary-antibodies/goat-mouse-igg-hl-alexa-fluor-647-ab150115.html
https://www.thermofisher.com/antibody/product/Donkey-anti-Rabbit-IgG-H-L-Highly-Cross-Adsorbed-Secondary-Antibody-Polyclonal/A-21206
https://www.abcam.com/products/secondary-antibodies/donkey-rabbit-igg-hl-alexa-fluor-594-preadsorbed-ab150064.html
https://www.thermofisher.com/antibody/product/CD45-Antibody-clone-30-F11-Monoclonal/53-0451-82
https://www.scbt.com/p/aqp1-antibody-1-22
https://www.bdbiosciences.com/en-eu/products/reagents/flow-cytometry-reagents/research-reagents/single-color-antibodies-ruo/apc-rat-anti-mouse-cd8a.561093
https://www.thermofisher.com/antibody/product/CD335-NKp46-Antibody-clone-29A1-4-Monoclonal/16-3351-81
https://www.cellsignal.com/products/primary-antibodies/doublecortin-antibody/4604
https://www.abcam.com/products/primary-antibodies/tmem119-antibody-28-3-microglial-marker-ab209064.html
https://www.biolegend.com/en-us/products/purified-anti-tubulin-beta-3-tubb3-antibody-11579?GroupID=GROUP686

Eukaryotic cell lines

Policy information about cell lines and Sex and Gender in Research

Cell line source(s)

Isolation of mouse primary microglia was done post-natal day 1-2 from a complete litter, thus the cells are from both sexes. VeroB4 cells (Lindqvist et al, J Neuroinflammation, 17, 284 (2020))were used for generating viral stock that was used to infect the animals and microglia cultures. VeroB4 were also used to titrate the virus so that correct amount of virus were used during infection.

Authentication

Microglia marker Iba1 was used to validate the purity of the primary cell.

Mycoplasma contamination

The cells were not contaminated with mycoplasm tested by MycoAlert (Lonza, Cat # LT07-318)

Commonly misidentified lines (See ICLAC register)	No commonly misidentified lines used in these experiments

Animals and other research organisms

Policy information about studies involving animals; ARRIVE guidelines recommended for reporting animal research, and Sex and Gender in Research

Laboratory animals	We used WT mice and Ifnar–/– mice in C57BL/6 background. The animals included in the experiments were 7-13 week-old and we include both sexes. Mice were house in individually ventilated cages with 21oC ± 1 oC ambient temperature, 55 ± 5% humidity and 12 hour light/dark cycle (6:00 am-6:00 pm).
Wild animals	No wild animals used in these experiments
Reporting on sex	We did not observe sex-based differences from our previous works. Both sexes were included in this study. Statements indicating mixed gender or a pool of male and female brains were included in the Materials and methods section.
Field-collected samples	No field-collected samples used in these experiments
Ethics oversight	Animal experiments were approved by the regional Animal Research Ethics Committee of Northern Norrland and by the Swedish Board of Agriculture (ethical permits: A9-2018 and A41-2019), and all procedures were performed according to their guidelines

Note that full information on the approval of the study protocol must also be provided in the manuscript.

Flow Cytometry

Plots

Confirm that:

☒ The axis labels state the marker and fluorochrome used (e.g. CD4-FITC).

☒ The axis scales are clearly visible. Include numbers along axes only for bottom left plot of group (a 'group' is an analysis of identical markers).

☒ All plots are contour plots with outliers or pseudocolor plots.

☒ A numerical value for number of cells or percentage (with statistics) is provided.

Methodology

Sample preparation	One sample were prepared from cerebral cortex from both hemispheres of one mouse and the non-neuronal cells purified by percoll gradient without fixation. Mixed gender animals were used in all experiments.
Instrument	ZE5 Cell Analyzer (Bio-Rad)
Software	FlowJo v10.0.7r2
Cell population abundance	Cell population abundance of microglia and brain leukocytes was calucaled as percentage of parent (single cells).
Gating strategy	Cells were first gated based on their size and granularity (FSC-area/SSC-area), then putative single cells were selected based on the relationship between area and height (FSC-area/FSC-height). Finally, populations of microglia and brain leukocytes were gated based on their expression of respective markers as indicated in exemplifying figures.

☒ Tick this box to confirm that a figure exemplifying the gating strategy is provided in the Supplementary Information.

Magnetic resonance imaging

Experimental design

Design type	n/a (no functional MRI)
Design specifications	n/a
Behavioral performance measures	n/a

Acquisition

Imaging type(s): Structural

Field strength: 9.4T

Sequence & imaging parameters: T1 Modified Driven Equilibrium Fourier Transform (MDEFT) with 5 repetitions (TR: 3000 ms; TE: 3ms; TI: 950 ms) acquired using a cryogenic RF coil. PMV matrix: 400x320x200. FOV:1.6x1.28x0.8 cm. Voxel size 0.04x0.04x0.04mm

Area of acquisition: ex vivo whole brain

Diffusion MRI: ☐ Used ☒ Not used

Preprocessing

Preprocessing software: Paravision 7.0 dcm2nii plugin MRIcron. SPM 8.

Normalization: n/a

Normalization template: n/a

Noise and artifact removal: n/a

Volume censoring: n/a

Statistical modeling & inference

Model type and settings: n/a

Effect(s) tested: n/a

Specify type of analysis: ☒ Whole brain ☐ ROI-based ☐ Both

Statistic type for inference (See Eklund et al. 2016): n/a

Correction: n/a

Models & analysis

n/a	Involved in the study
☒	☐ Functional and/or effective connectivity
☒	☐ Graph analysis
☒	☐ Multivariate modeling or predictive analysis
